# Anthocyanin-rich blue potato meals protect against polychlorinated biphenyl-mediated disruption of short-chain fatty acid production and gut microbiota profiles in a simulated human digestion model

**DOI:** 10.3389/fnut.2023.1130841

**Published:** 2023-05-31

**Authors:** Fang Lu, Chad W. MacPherson, Julien Tremblay, Michèle M. Iskandar, Stan Kubow

**Affiliations:** ^1^School of Human Nutrition, McGill University, Sainte-Anne-de-Bellevue, QC, Canada; ^2^NutraPharma Consulting Services, Inc., Montreal, QC, Canada; ^3^Energy, Mining and Environment, National Research Council Canada, Montreal, QC, Canada

**Keywords:** polychlorinated biphenyl 153, polychlorinated biphenyl 126, gut microbiota, anthocyanins, short-chain fatty acids, simulated gut model, 16S rRNA gene amplicon sequencing, V3–V4 hypervariable regions

## Abstract

**Background:**

Polychlorinated biphenyls (PCBs) are ubiquitous environmental pollutants associated with a wide variety of adverse human health outcomes. PCB 126 and PCB 153 are among the most prevalent congeners associated with human exposure. Emerging studies have suggested that PCB exposure leads to lower gut microbial diversity although their effects on microbial production of health promoting short-chain fatty acids (SCFAs) has been scarcely studied. Blue potatoes are rich in anthocyanins (ACNs), which is a class of polyphenols that promote the growth of beneficial intestinal bacteria such as *Bifidobacterium* and *Lactobacillus* and increase the generation of SCFAs. A batch-culture, pH-controlled, stirred system containing human fecal microbial communities was utilized to assess whether human gut microbiota composition and SCFA production are affected by: (a) PCB 126 and PCB 153 exposure; and (b) ACN-rich digests in the presence and absence of the PCB congeners.

**Methods:**

Anthocyanin-rich blue potato meals (11.03 g) were digested over 12 h with and without PCB 126 (0.5 mM) and PCB 153 (0.5 mM) using an *in vitro* simulated gut digestion model involving upper gastrointestinal digestion followed by metabolism by human fecal microbiota. Fecal digests were collected for analysis of gut microbial and SCFA profiles.

**Results:**

Polychlorinated biphenyl-exposed fecal samples showed a significant (*p* < 0.05) decrease in species richness and a significantly (*p* < 0.05) different microbial community structure. PCB treatment was associated with an increased (*p* < 0.05) relative abundance of *Akkermansia, Eggerthella*, and *Bifidobacterium* and a decreased (*p* < 0.05) relative abundance of *Veillonella, Streptococcus*, and *Holdemanella*. ACN digests counteracted the altered abundances of *Akkermansia* and *Bifidobacterium* seen with the PCB treatment. PCB exposure was associated with a significant (*p* < 0.05) decrease in total SCFA and acetate concentrations. ACN digests were associated with significantly (*p* < 0.05) higher SCFA and acetate concentrations in the presence and absence of PCBs.

**Conclusion:**

Human fecal matter exposed to PCB 126 and PCB 153 led to decreased abundance and altered gut microbiota profiles as well as lowered SCFA and acetate levels. Importantly, this study showed that prebiotic ACN-rich potatoes counteract PCB-mediated disruptions in human gut microbiota profiles and SCFA production.

## 1. Introduction

Polychlorinated biphenyls (PCBs) are a class of ubiquitous persistent organic pollutants consisting of 209 congeners. They can be categorized into dioxin-like and non-dioxin-like PCBs based on the number and the substitution sites of chlorine atoms on the biphenyl rings (1). Although PCBs have been banned since the last century owing to their environmental and human toxicity, they are still present at measurable levels in the environment and in human tissues and plasma (2–4). Their high lipophilicity and resistance to biodegradation enable PCBs to bioaccumulate and biomagnify throughout the food chain, making food consumption the primary route of human exposure to PCBs (5, 6). PCB 126 and PCB 153 are among the most potent dioxin-like and non-dioxin-like PCBs, respectively (1). These two congeners are predominant congeners found in food (7) and in human plasma (2). The plasma level of ∑PCBs is 0.635 μg/L in the Canadian general population (4). The PCB exposure disrupts host metabolism (8, 9) and increases the risk of metabolic disorders including obesity, type 2 diabetes and liver diseases (10–12). Chronic inflammation is one of the key features of these metabolic disorders (13). Data from animal model studies and *in vitro* studies have reported an association between PCB exposure and inflammation (14–16).

The gut microbiota consists of trillions of microorganisms contributing to xenobiotic, nutrient, and energy metabolism (17, 18). External influences such as environmental pollutants and diet can alter gut microbiota composition and structure (19, 20). Loss of gut microbiota abundance or imbalance in gut microbiota structure, which is referred to as gut microbiota dysbiosis, is associated with the development of metabolic disorders including obesity and type 2 diabetes (19, 21). Microbial metabolites are one of the crucial mediators of gut microbiota and host communication (22). In that regard, short-chain fatty acids (SCFAs), especially acetate, propionate and butyrate, are major microbial metabolites produced by gut bacterial fermentation of non-digestible dietary polysaccharides (22). SCFAs are key mediators involved in the effects of gut microbiota on intestinal homeostasis and inflammation (22, 23). For instance, decreased abundance of butyrate-producing bacteria and butyrate depletion have been associated with impaired gut barrier function and chronic inflammation in patients with inflammatory bowel disease (24, 25). The host-related benefits of SCFAs include controlling inflammation, maintaining gut barrier integrity and beneficially modulating the gut microbiota composition (26).

Emerging animal studies have demonstrated that PCB exposure reduces the diversity of gut microbiota and disrupts their community composition (14, 27–29). Mouse models have shown that PCB 126 exposure is associated with decreased levels of *Firmicutes* (28) and increased levels of gram-negative bacteria such as *Bacteroidetes* (28, 30). Furthermore, PCB treatment was associated with elevated levels of lipopolysaccharides in the peripheral circulation and upregulated proinflammatory cytokines in the mouse brain (30). Non-dioxin-like PCB 153 exposure was linked to the reduced diversity of gut microbiota as well as to the increased abundance of *Firmicutes* and reduced abundance of *Bacteroidetes* (29). Notably, there is scarce information regarding the impact of PCBs on the microbial production of SCFAs. A recent study has shown that PCB 126 exposure in adult mice lowered cecal SCFA concentrations (31). Conversely, an increased abundance of SCFA-producing bacteria *Roseburia* and *Ruminiclostridium* was shown in mice exposed to a PCB congener mixture, although gut SCFA profiles were not studied (32).

Prebiotics are non-digestible food components that improve host health by stimulating the growth or activity of colonic bacteria (33). To date, no previous studies have assessed whether PCB-induced gut microbiota dysbiosis could be counteracted by prebiotics. Anthocyanins (ACNs), which are pigments found in the skin and flesh of colored plant foods, have prebiotic activity (34). The major dietary sources of ACNs are fruits and vegetables (35). Besides well-known ACN-rich foods such as berries, blue and purple fleshed potatoes have a rich anthocyanin content (36). The primary ACNs in blue and purple fleshed potatoes are petunidin 3-p-coumaroylrutinoside-5-glucoside and malvidin 3-feruloylrutinoside-5-glucoside (37). Due to the low bioavailability of ACNs, the majority of ACNs reach the colon and interact with gut microbiota (38). Previous results have demonstrated beneficial effects of ACNs on the gut microbiota community structure and metabolic functions including the production of SCFAs (38–40). For instance, Jennings et al. (41) showed that participants with a higher dietary ACN intake had a greater microbiota diversity and an increased abundance of butyrate-producing bacteria, including *Clostridiales* and *Ruminococcaceae* in a cross-sectional population study. ACN extracts from purple sweet potatoes have been shown to increase total SCFA and acetate production after 24 h *in vitro* fermentation with human fecal matter (40). The extracts also increased the relative abundance of *Bifidobacterium, Lactobacillus-Enterococcus*, and reduced the relative abundance of *Bacteroides-Prevotella* and *Clostridium* (40).

The computer-controlled batch culture fermentation system is a validated and convenient model to assess the short-term impact of dietary components such as polyphenols on gut microbiota profile and production of microbial metabolites such as SCFAs (42). This closed fermentation system utilizes a sealed reactor sequentially representing the oral to intestinal digestion, followed by colonic fermentation with fecal microbiota. The present study utilized this batch culture fermentation system containing human fecal microbiota to assess whether gut microbiota composition and SCFA production was affected by treatment with: (a) PCB congeners 153 and 126; and (b) ACN-rich potato meals (11.03 g) in the presence and absence of the two PCB congeners.

## 2. Materials and methods

### 2.1. ACN-rich potato meal preparation

The All Blue (Russian Blue) purple-fleshed potato cultivar was used as the source of ACNs. Purple-fleshed potatoes have a higher ACN content compared to other colored potatoes (43, 44). Two kilograms of organic All Blue potato tubers, with purple skin and flesh, were obtained from West Coast Seeds (Vancouver, BC). Potatoes were washed with water, cut into small cubes and cooked at 100^°^C for 20 min. After cooling at room temperature, cooked potatoes were loosely packed and stored at −20^°^C until freeze-drying. All samples were freeze-dried at −50 to −60^°^C with the vacuum pressure at 0.85 mbar (0.64 mm Hg) using a Christ LCG Lyo Chamber Guard freeze dryer (Gamma 1-16 LSC, Osterode am Harz, Germany) under previously established conditions (45). The freeze-dried potato cubes were ground (CBG100SC, Black and Decker, Towson, MD, USA) and stored at −80^°^C until further use.

### 2.2. Simulated gastrointestinal (GI) fermentation

**FIGURE 1:**
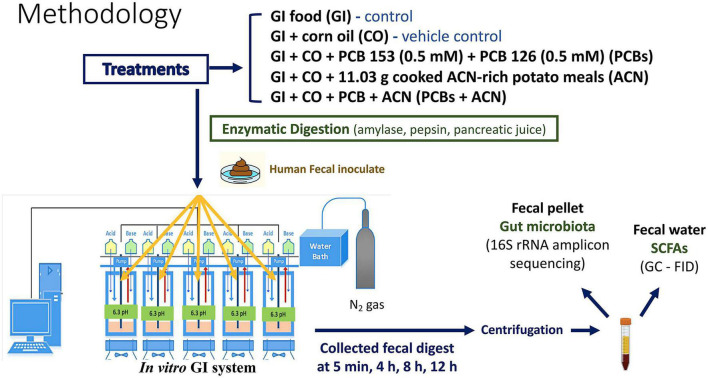
Workflow overview. Polychlorinated biphenyl (PCB) 153 and PCB 126, with or without anthocyanins (ACNs), along with negative control and vehicle control were subjected to the batch culture fermentation system, followed by sample collection. Samples were centrifuged, and pellets were collected for 16S rRNA amplicon sequencing. Supernatants (fecal water) were analyzed via gas chromatography-flame ionization detection (GC-FID) to determine short-chain fatty acid (SCFA) concentrations.

This study used a dynamic computer-controlled batch culture fermentation system to simulate the oral to colonic conditions (46, 47). A total of five fermentation reactors (200 mL, one per treatment) were run in parallel. The physiological conditions from oral to colonic, including temperature, pH and anaerobic environment were strictly controlled. The temperature of each reactor was maintained at 37^°^C throughout the digestion by a circulating water bath. The pH was controlled continuously by an embedded EZO™ pH circuit (Atlas Scientific, Long Island City, NY, USA) through a Raspberry Pi microprocessor (ver. 1B Raspberry Pi Foundation, Cambridge, UK). The anaerobic condition of each reactor was maintained by oxygen-free nitrogen gas. The workflow overview of this study is shown in Figure 1.

### 2.3. Batch culture fermentation

On the day of the experiment, each reactor was filled with 90 mL of gastrointestinal (GI) food that consisted of arabinogalactan (1 g/L), pectin (2 g/L), glucose (0.4 g/L), xylan (1 g/L), starch (3 g/L), mucin (4 g/L), proteose peptone (1 g/L), yeast extract (3 g/L), L-cysteine (0.5 g/L), NaHCO_3_ (0.4 g/L), Tween 80 (1 mL/L), vitamin solution (40 μL/L) (Sigma Aldrich, St. Louis, MO, USA) and autoclaved distilled water. The pH of GI food was adjusted to seven before use. The formula of GI food was based on Molly et al. (48), which has been shown to provide optimal growth conditions for commensal gut bacteria.

#### 2.3.1. Preparation of treatments

Five treatments were prepared and 90 mL GI food was added to each reactor according to the following treatments: GI control (2 mL GI food), corn oil vehicle control (CO) (2 mL corn oil; No Name, Loblaws Inc., Toronto, ON, Canada), ACN-rich potato meal treatment (ACN) (11.03 g of freeze-dried organic All Blue potato powder with 2 mL corn oil), PCB treatment (1 mL of 1 mM PCB 126 and 1 mL of 1 mM PCB 153 dissolved in corn oil; PCB 126 Cat# P274493, PCB 153 Cat# H290835, Toronto Research Chemicals, North York, ON, Canada), and ACN-rich potato meal with the PCB co-treatment (PCBs+ACN) (11.03 g of freeze-dried organic All Blue potato powder with 2 mL PCB mixture). The dosage of PCBs exposed to the fecal samples (5 μM ) was equivalent to the PCB dosage in plasma (3.4 μM) from acute PCB-exposed individuals (49). Corn oil was the vehicle control used to dissolve the lipophilic PCBs instead of dimethyl sulfoxide that has anti-bacterial effects (50). The ACN content of purple-fleshed potatoes has a range of 40.5–46.5 mg/100 g fresh weight (36, 51). Raw and boiled potatoes have a water content of 77–80 and 20–23% of dry matter, respectively (52). In the present study, 11.03 g freeze dried, purple-fleshed potato powder was added into the batch culture fermentation system, which provides an ACN content 18–22 mg. The estimated daily intake of ACNs in the United States and Australia is 12.5 mg/day/person and 24.2 mg/day/person, respectively. Hence, the ACN content (18–22 mg) in this study is dietarily relevant.

#### 2.3.2. Upper GI digestion

The upper GI digestion procedure was adapted from Tzounis et al. (47) and Gaisawat et al. (53). Oral digestion was performed by addition of α-amylase (2 mL 0.47 g/mL; A3176, Sigma Aldrich, St. Louis, MO, USA) with 5 min incubation at 37^°^C and pH 7.0, followed by adjusting the pH to 2 and adding pepsin solution (2 mL 0.8 g/mL; P7125, Sigma Aldrich, St. Louis, MO, USA) to simulate gastric digestion at 37^°^C for 1.5 h. Subsequently, the pH was adjusted to eight using NaOH (1N, Sigma Aldrich, St. Louis, MO, USA) and then 30 mL pancreatic juice [NaHCO_3_ (12 g/L), bile extract (6 g/L) and pancreatin (0.9 g/L), Sigma Aldrich, St. Louis, MO, USA] were added and incubated at 37^°^C for 2 h to mimic small intestinal digestion.

#### 2.3.3. Fecal slurry preparation

Fecal samples were collected from a healthy, non-smoking 26-year-old female volunteer who had not taken antibiotics for at least 6 months before the study. Fecal samples were collected 3 days before the experiment and were aliquoted and frozen according to Gaisawat et al. (46). Briefly, 211.09 g fecal samples were collected in the morning and mixed with 12.5% glycerol with 0.9% saline at a ratio of 1:4 v/v. Samples were then filtered using Whirl-PAK sterile filter bags (B01348WA, Thermo Fisher Scientific, Pittsburgh, PA, USA), aliquoted and stored at −80^°^C until further use. Frozen fecal samples (200 mL) were pre-stabilized at 37^°^C under anaerobic conditions for 12 h prior to batch culture fermentation. Fecal samples were defrosted at room temperature and transferred into pre-sterilized bottles containing 160 mL GI food using syringes.

#### 2.3.4. Fermentation

After the completion of pancreatic digestion, the digesta were exposed to reactors that were inoculated with pre-stabilized fecal slurry (80 mL) to initiate the fermentation (T = 5 min). After inoculation, fermentation was carried out for 12 h under anaerobic conditions with the pH controlled at 6.8. Fecal water samples (6 mL) were collected every 4 h after which they were centrifuged at 2,000 g for 20 min. The supernatant was filtered through 0.2 μm filters and stored at −20^°^C for later analysis. The pellets were stored at −80^°^C for further extraction.

### 2.4. SCFA analysis

Short-chain fatty acids were separated and detected using a 6,890 series gas chromatograph system equipped with a flame ionization detector (GC-FID) (Agilent Technologies, Santa Clara, CA, USA), based on a modified method by Sadeghi Ekbatan et al. (54). Briefly, 100 μL samples were filtered using 0.45 μm syringe filters with 1 μL of filtered sample directly injected into the GC-FID equipped with a fused capillary column (30 m × 250 μm ID × 0.25 μm file thickness, HP-INNOWAS, Agilent Technologies, Santa Clara, CA, USA). The SCFAs were separated using a HP-INNOWAS 30 m fused capillary column with 250 μm internal diameter and a film thickness of 0.25 μm. Helium was used as the carrier gas at a flow rate of 1 mL/min. The inlet and detection temperatures were set at 220 and 230^°^C, respectively. The oven temperature was initially set at 100^°^C, held for 2 min and then raised 10^°^C/min until 220^°^C and held for 1 min. The SCFAs were identified and quantified based on their retention times compared with free volatile fatty acid standards at concentrations ranging from 0 to 10 mM (46975-U, Sigma Aldrich, St. Louis, MO, USA). The concentrations of individual and total SCFAs were calculated in mM. Samples were analyzed in duplicate from each run of the batch culture fermentation.

### 2.5. DNA extraction

Total DNA was isolated from approximately 250–300 mg fecal pellets from each run of the batch culture fermentation using the QIAamp Fast DNA Stool Mini Kit (51604, Qiagen, Hilden, Germany) according to the manufacturer’s protocol with modifications. The fecal samples were washed twice with 0.05 M phosphate buffer and incubated at 70^°^C with 1 mL InhibitEX (Qiangen, Hilden, Germany) before adding 0.1 mm zirconia/silica beads (∼300 mg/tube; 360991112, Thermo Fisher Scientific, Pittsburgh, PA, USA) to each sample tube. Samples were then homogenized three times using a bead-beater (MP FastPrep-24, MP Biomedicals, Irvine, CA, USA) at 4 m/s for 1 min prior to centrifugation of samples at 9,000 g for 3 min to remove the fecal particles. DNA purity was assessed by 260/280 ratio; the ratios obtained for all DNA samples were between 1.6 and 2.7 and were used for 16S rRNA gene amplicon sequencing.

### 2.6. 16S rRNA gene amplicon sequencing

The extracted DNA amplification and sequencing followed the method described by MacPherson et al. (55). The sequencing library was prepared according to Illumina’s “16 S Metagenomic Sequencing Library Preparation Guide” (Part # 15044223 Rev. B) with the exception of the use of Qiagen HotStar MasterMix for the first polymerase chain reactions (PCR) (amplicon PCR) and halving reagent volumes for the second PCR. The 16S V3–V4 hypervariable regions were amplified based on the following primers (without the overhang adapter sequence): 5′-CCTACGGGNGGCWGCAG-3′ (forward) and 5′- GACTACHVGGGTATCTAATCC-3′ (reverse), generating a fragment of around 460 bp. The first PCR (amplicon PCR) was carried out for 25 cycles with annealing temperature of 55^°^C. Diluted pooled samples were loaded on an Illumina MiSeq system and sequenced using a 500-cycle (paired-end sequencing configuration of 2 × 250 bp) MiSeq Reagent Kit v3.

### 2.7. Microbiota sequencing data analysis

Sequencing data was analyzed using AmpliconTagger (56). Briefly, raw reads were scanned for sequencing adapters and PhiX spike-in sequences. Remaining reads were removed that failed to meet one of the following conditions: having average quality Phred score lower than 25; having 40 bases of quality lower than Phred score 15; and having no undefined bases (N). The remaining sequences were processed for generating Amplicon Sequence Variants (ASVs) (DADA2 v1.12.1) (57). Since a quality filtering step was performed in a separate upstream step (described above), we used more lenient parameters for the DADA2 workflow which is summarized as follows: filterAndTrim (maxEE = 2, truncQ = 0, maxN = 0, minQ = 0). Errors were learned using the learnErrors (nbases = 1e8) function for both forward and reverse filtered reads. Reads were merged using the mergePairs (minOverlap = 12, maxMismatch = 0) function. Chimeras were removed with DADA2’s internal removeBimeraDeNovo (method = “consensus”) method followed by UCHIME reference (58). ASVs were assigned a taxonomic lineage with the RDP classifier (59) using an in-house training set (doi: 10.5281/zenodo.356015) containing the complete SILVA release 128 database (60) supplemented with eukaryotic sequences from the SILVA database and a customized set of mitochondria, plasmid and bacterial 16S sequences. The RDP classifier gave a score (0–1) to each taxonomic depth of each ASV. Each taxonomic depth having a score ≥0.5 was kept, reconstructing the final lineage. Taxonomic lineages were combined with the ASV abundance matrix obtained above to generate a raw ASV table, from which a bacterial organisms ASV table was generated. Five hundred 1,000 reads rarefactions were then performed on this latter ASV table and the average number of reads of each ASV of each sample was computed to obtain a consensus rarefied ASV table. A phylogenetic tree (needed to compute weighted UniFrac distances) was obtained by aligning ASV sequence representatives on a Greengenes core reference alignment (61) using the PyNAST v1.2.2 aligner (62). Alignments were filtered to keep only the hypervariable region of the alignment. A phylogenetic tree was then built from that alignment with FastTree v2.1.10 (63). Alpha (Chao 1 index, Shannon index and observed species) and beta (weighted, unweighted UniFrac and Bray Curtis distances) diversity metrics and taxonomic summaries were then computed using the QIIME v1.9.1 software suite (62, 64) using the consensus rarefied OTU table and phylogenetic tree (i.e., for UniFrac distance matrix generation). In total, 745 bacterial ASVs were identified.

### 2.8. Statistical analysis

All data are shown as means ± standard error of the mean (SEM). SCFA data and gut microbiota beta diversity were analyzed using two-way ANOVA with treatments and time as factors followed by Tukey’s HSD multiple comparisons test. When a significant interaction between treatments and time was observed, the means of each treatment within the same timepoint were compared. When the interaction between two factors was not significant, the means of all timepoints were jointly considered. Statistical analyses used JMP v14.2 and statistical visualizations for SCFAs and beta diversity used GraphPad Prism 9.

## 3. Results

### 3.1. Gut microbiota diversity and composition

**FIGURE 2:**
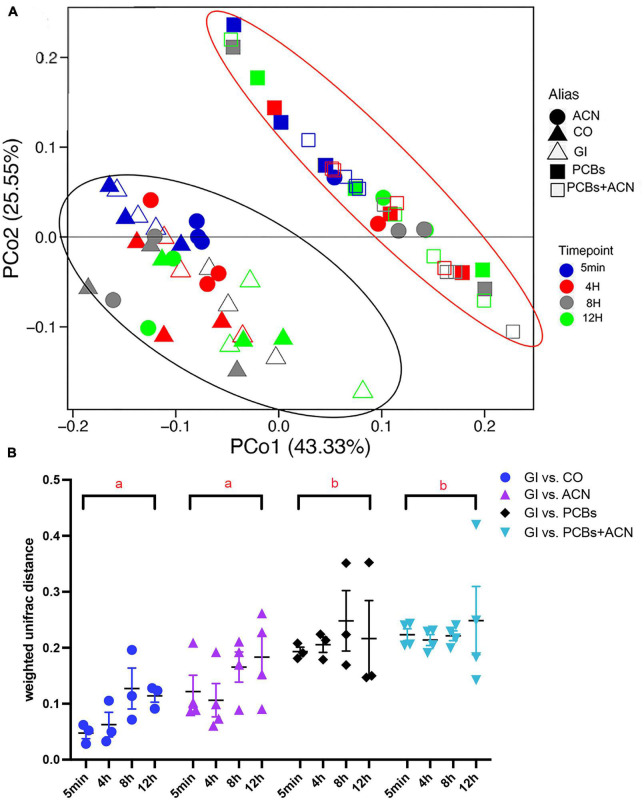
Beta diversity of gut microbiota showing: **(A)** principal coordinate analysis (PCoA) plot clustered by different treatments. The red ellipse highlights the clustering of polychlorinated biphenyl (PCB)-containing treatments and the black ellipse, the clustering of non-PCB-containing treatments; and **(B)** weighted UniFrac distance comparing each treatment (PCBs, ACN, and PCBs+ACN) with controls (GI vs. CO) at corresponding digestion timepoints. Treatments not sharing a letter (a, b) have significantly different weighted UniFrac distances with all timepoints jointly combined (*p* < 0.05) {*n* = 3 [gastrointestinal (GI), corn oil (CO), and PCBs], *n* = 4 (ACN and PCBs+ACN)}.

Beta diversity was used to compare the similarity of gut microbiota profiles between each treatment and controls over the 12 h digestion (Figure 2). The principal coordinate analysis (PCoA) plot shows two distinct separations between PCB-containing groups (PCBs and PCBs+ACN) and controls (GI and CO) (Figure 2A). The ACN alone treatments tended to cluster with the GI and CO treatments, except for one replicate from the 4 h ACN alone digestion (one red dot), two replicates from the 8 h ACN alone digestion (two gray dots) and one replicate from the 12 h ACN alone digestion (one green dot), which clustered with samples from the PCBs alone and PCBs+ACN treatments. However, these data points did not meet the conditions to be statistically considered outliers. Therefore, we also represented the Weighted Unifrac data in a dot plot (Figure 2B) with a two-way ANOVA followed by Tukey’s HSD test. The weighted UniFrac distance, which accounts for the relative abundance of each taxon within the communities, was used to assess the similarity of microbiota profiles among the different treatments over time. Two-way ANOVA showed only a significant (*p* < 0.05) main effect of treatment (Figure 2B). When the means of all time points were jointly considered, the PCB treatments (PCBs vs. GI; PCBs+ACN vs. GI) had more distant microbiota community profiles as compared to non-PCB containing treatments (GI vs. CO; ACN vs GI), indicating that the PCBs altered gut microbiota community structure. It appears that ACN treatment could not protect against the PCB-induced alteration of gut microbiota community structure as PCBs+ACN had a higher UniFrac distance compared to ACN vs. GI and controls. There were no significant differences in weighted UniFrac distances between ACN vs. GI and control, indicating that ACNs had a similar microbiota community structure as controls.

**FIGURE 3:**
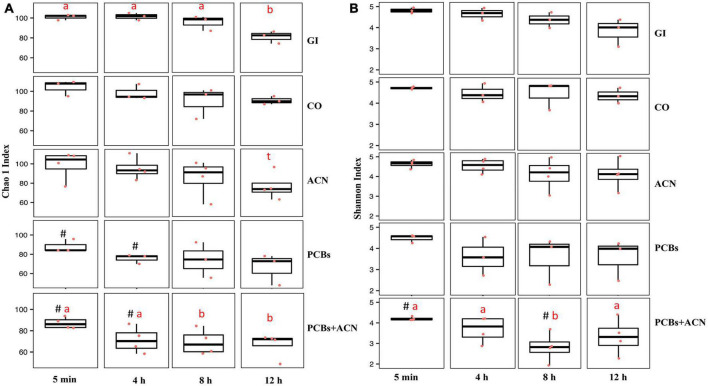
Alpha diversity of gut microbiota quantified by the Chao 1 index for species richness **(A)** and Shannon index for species richness and evenness **(B)** for the five treatments at 5 min, 4, 8, and 12 h of digestion. The means at different time points within the same treatment not sharing a letter (a, b) are significantly different (*p* < 0.05). The symbol # represents a significant difference (*p* < 0.05) between treatment and vehicle control corn oil (CO) at corresponding digestion time points. The symbol t represents a trend (*p* = 0.07) over 12 h digestion within the same treatment {*n* = 3 [gastrointestinal (GI), CO, and polychlorinated biphenyls (PCBs)], *n* = 4 [anthocyanin (ACN) and PCBs+ACN]}.

Alpha diversity was used to determine the impact of PCBs and ACNs on the richness and evenness of the gut microbiota community (Figure 3). Chao 1 index (65) assessed the species richness within treatment at 4, 8, and 12 h versus the control digestion at 5 min (Figure 3A). The species richness of both the CO and PCB treatments was not significantly changed over time. A trend (*p* = 0.07) for decreased trend of species richness was shown at 12 h of digestion with the ACN treatment. Species richness was significantly (*p* < 0.05) reduced in the GI control at 12 h and in the PCBs+ACN treatment at 8 and 12 h indicating that species richness was not well-maintained over the 12 h digestion.

The ACN treatment showed no significant differences in Chao 1 index (*p* > 0.05) across time compared to CO at corresponding time points indicating no change in species richness over the 12 h digestion. In view of the decreased species richness in the GI group, the effects of PCBs and ACN treatments on alpha diversity and uniformity using the Shannon index (66) were compared to the vehicle CO control at each time point (Figure 3B). In terms of microbial community diversity and evenness assessed by the Shannon index, the PCBs treatment showed no differences as compared to CO at the corresponding time points over the 12 h digestion. A decrease in the Shannon index between PCBs+ACN compared to the CO control was shown at the beginning of the digestion at 5 min (*p* < 0.05) and at 8 h of digestion (*p* < 0.05). The ACN treatment showed no significant differences in species diversity (*p* > 0.05) across time compared to that of vehicle control CO at all time points. In terms of the changes in species diversity within a given treatment, only PCBs+ACN showed a decreased Shannon index (*p* < 0.05) at 8 h compared to the Shannon index at 5 min of digestion in the same treatment.

**FIGURE 4:**
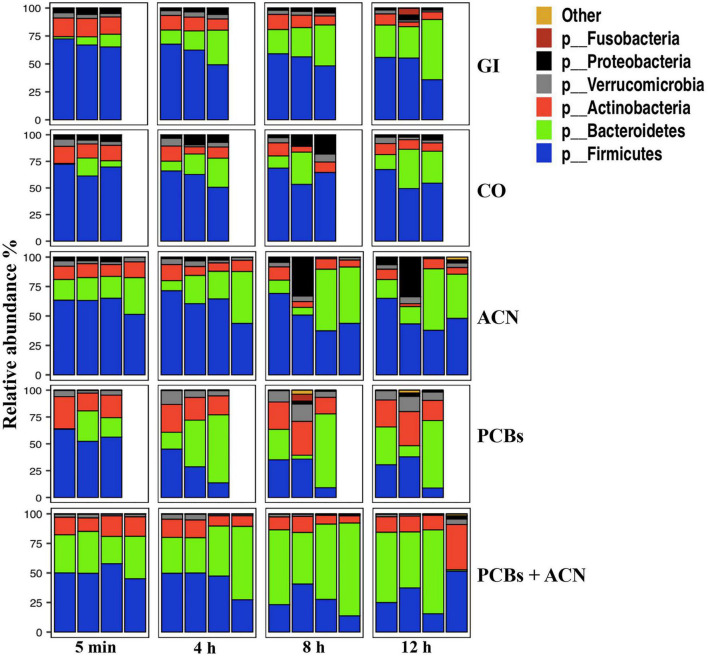
Taxonomic profiles of the top six taxa down to the phylum level of fecal samples collected from the *in vitro* gastrointestinal (GI) model over 12 h digestion. The gut microbiota profile under different treatment groups represents the relative microbiota composition abundance in Amplicon Sequence Variant (ASV) for each fermentation replicate {*n* = 3 [GI, corn oil (CO), and polychlorinated biphenyls (PCBs)], *n* = 4 [anthocyanin (ACN) and PCBs+ACN]}.

**FIGURE 5:**
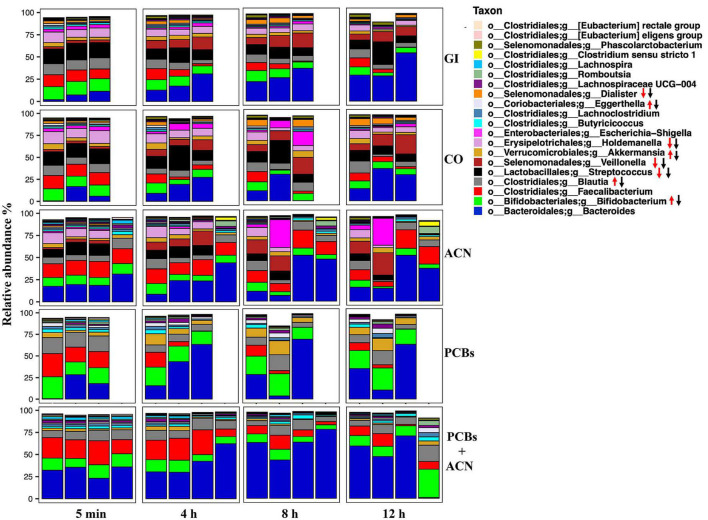
Taxonomic profiles of the top 20 taxa down to the genus level of fecal samples collected from the *in vitro* gastrointestinal (GI) model over 12 h digestion. The gut microbiota profile under different treatment groups represents the relative microbiota composition abundance in Amplicon Sequence Variant (ASV) for each fermentation replicate. Red arrows represent taxa regulated by polychlorinated biphenyls (PCBs), black arrow represents taxa regulated by PCBs+ACN {*n* = 3 [GI, corn oil (CO), and PCBs], *n* = 4 [anthocyanin (ACN) and PCBs+ACN]}.

The relative abundance of the top six taxa down to the phylum level and the top 20 taxa down to the genus level of the fecal samples collected from the GI model are shown in Figures 4, 5. The dominant taxa at the phylum level were *Firmicutes, Bacteroidetes*, and *Actinobacteria* for all treatments over time while *Verrucomicrobia* and *Proteobacteria* were the least dominant phyla for all treatments. Figure 4 shows that PCB exposure reduced the relative abundance of *Proteobacteria* and *Firmicutes* while the relative abundance of *Verrucomicrobia* and *Actinobacteria* was increased over time. PCBs+ACN also reduced the relative abundance of *Proteobacteria* and *Firmicutes* but the relative abundance of *Bacteroidetes* was increased compared to the GI and CO controls over the 12 h period. The dominant taxa at the phylum level were relatively similar between ACNs and the GI and CO controls over the 12 h digestion time.

At the genus level, ACNs had a similar taxonomic profile compared to controls whereas PCBs and PCBs+ACN showed a different taxonomic profile compared to the GI and CO controls over the 12 h digestion time (Figure 5). The predominant taxa at the genus level were *Bacteroides, Bifidobacterium*, *Faecalibacterium, Blautia, Streptococcus*, and *Holdemanella* in GI, CO, and ACN treatments at 5 min of digestion. The less dominant taxa in GI, CO, and ACN samples included *Veillonella, Akkermansia, Butyricicoccus*, and *Dialister* at 5 min of digestion. PCB exposure altered the taxonomic profile at the beginning of the digestion at 5 min. Specifically, the predominant taxa in PCBs were *Bacteroides, Bifidobacterium, Faecalibacterium*, and *Blautia* with the proportion of the relative abundance of *Blautia* increased compared to controls at 5 min of digestion. PCBs+ACN had a relatively similar taxonomic profile at the beginning of the digestion as PCBs. The predominant taxa were *Bacteroides, Bifidobacterium, Faecalibacterium*, and *Blautia* with a higher proportion of the relative abundance of *Bacteroides* and *Faecalibacterium* compared to control at 5 min of digestion.

The taxonomic profile changed during digestion with a decreased relative abundance of some taxa and enriched relative abundance of others for all treatments (Supplementary Figure 2). PCB treatment increased the relative abundance of *Akkermansia, Bifidobacterium, Blautia*, and *Eggerthella* and reduced the relative abundance of *Streptococcus, Veillonella, Holdemanella*, and *Dialister* over the 12 h digestion. The relative abundance of *Streptococcus, Veillonella, Holdemanella*, and *Dialister* were also reduced in PCBs+ACN; however, the co-treatment blunted the high relative abundance of *Bifidobacterium, Akkermansia*, and *Blautia* induced by PCBs, and also enriched the relative abundance of *Faecalibacterium* over the 12 h digestion. The ACN treatment showed an increased relative abundance of *Faecalibacterium* compared to controls.

### 3.2. Total and major individual SCFAs

**FIGURE 6:**
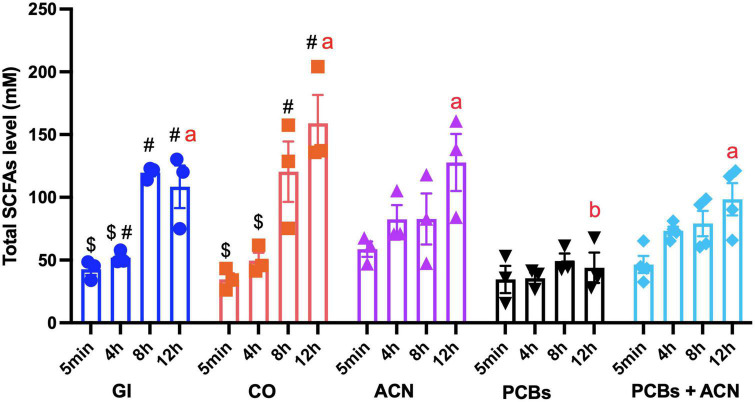
Total short-chain fatty acid (SCFA) concentrations with different treatments across time. Data is represented by means ± SEM. Two-way ANOVA followed by Tukey’s HSD test was used to assess for significant differences. Treatments not sharing common letters (a, b) are significantly different at the same digestion timepoints (*p* < 0.05). The symbols #, $ indicate significant differences (*p* < 0.05) over the 12 h digestion within the same treatment. Bars not sharing common symbols are statistically different between treatments (*p* < 0.05) {*n* = 3 [gastrointestinal (GI), corn oil (CO), anthocyanin (ACN), and polychlorinated biphenyls (PCBs)], *n* = 4 (PCBs+ACN)}.

**FIGURE 7:**
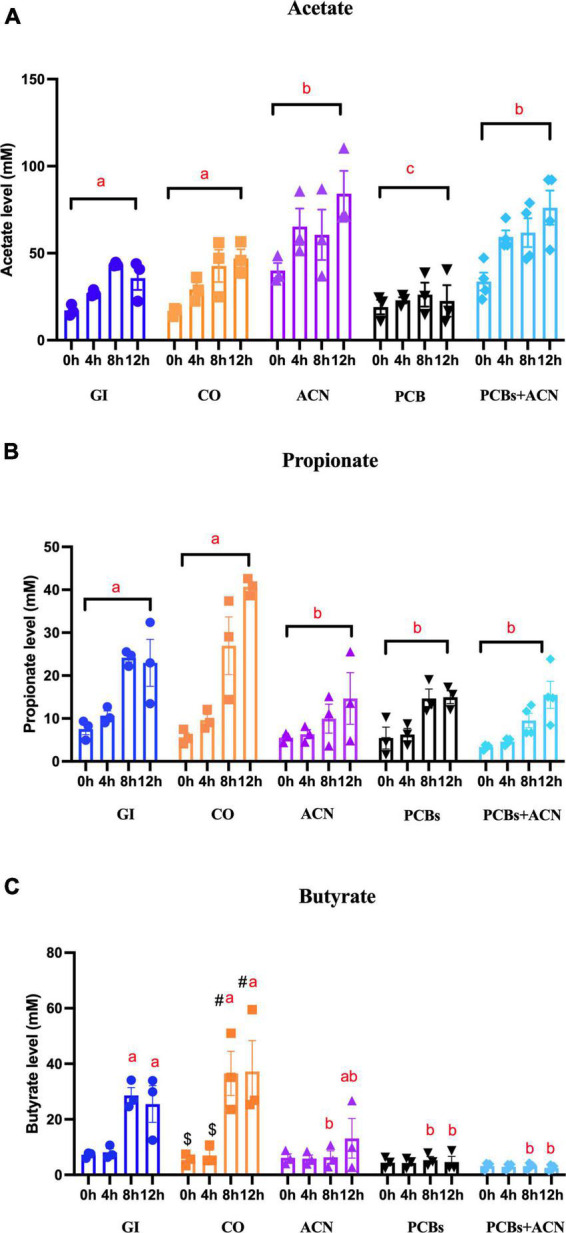
The concentrations of the three major individual short-chain fatty acids (SCFAs) [**(A)** acetate, **(B)** propionate, and **(C)** butyrate] in fecal water under different treatments. Data is represented by means ± SEM. Two-way ANOVA followed by Tukey’s HSD test was used to assess for significant differences. Treatments not sharing common letters (a, b, c) are significantly different (*p* < 0.05) at the same digestion timepoints. The symbols #, $ represent significant (*p* < 0.05) differences over the 12 h digestion time within the same treatment. Bars not sharing common symbols are statistically different between treatments (*p* < 0.05) {*n* = 3 [gastrointestinal (GI), corn oil (CO), anthocyanin (ACN), and polychlorinated biphenyls (PCBs)], *n* = 4 (PCBs+ACN)}.

Polychlorinated biphenyls significantly (*p* < 0.05) decreased total SCFA concentrations as compared to controls (GI and CO) at 12 h digestion (Figure 6). In contrast, the total SCFA level of the ACN and PCBs+ACN treatments showed no significant differences when compared to controls (GI and CO) at the 12 h digestion period. Acetate was the most abundant SCFA (Figure 7A). The PCBs group had a significantly (*p* < 0.05) lower acetate level as compared to the vehicle control CO with all timepoints jointly combined. ACN and PCBs+ACN had significantly (*p* < 0.05) higher acetate concentrations when compared to the vehicle control CO with all timepoints combined.

The PCBs treatment was associated with significantly (*p* < 0.05) lower propionate content (Figure 7B) as compared to controls with all timepoint jointly considered. The ACN and PCBs+ACN groups also had significantly (*p* < 0.05) reduced propionate content as compared to controls with all timepoints combined. PCBs was associated with reduced concentrations of butyrate (Figure 7C; *p* < 0.05) at 8 and 12 h digestion compared to controls at the corresponding timepoints. The ACN and PCBs+ACN treatments showed significantly (*p* < 0.05) reduced butyrate concentrations at the last two digestion timepoints compared to controls. The butyrate level in ACN alone had no significant difference between GI at 12 h digestion (*p* > 0.05) The concentrations of valeric acid, caproic acid, isocaproic acid and heptanoic acid ranged at concentrations around 3 mM (Supplementary Figure 1). Both PCBs and PCBs+ACN resulted in a lower concentration (*p* < 0.05) of these individual SCFAs compared to either GI control or CO control. No significant changes were noted in the concentration of isocaproic acid, valeric acid, caproic acid and heptanoic acids between ACN and control treatments (Supplementary Figure 1).

## 4. Discussion

The present findings demonstrate that PCBs disrupted human gut microbiota community structure and the abundance of specific taxa, as well as inhibited the production of SCFAs, particularly acetate. Specifically, PCBs significantly altered gut microbiota community structure by decreasing the gut microbial species richness and causing a shift in taxonomic profiles. The human fecal samples from the current study were exposed to digests containing 5 μM of PCB that mimics the plasma levels of PCBs in acutely exposed human populations (49). The observed PCB-induced alterations of gut microbiota structure in terms of beta and alpha diversity are consistent with animal model studies (14, 27–29). For instance, Min et al. (29) also demonstrated that PCB 153 exposure is associated with a decreased species richness and diversity in mice. They also observed that PCB 153 exposure resulted in a clear separation of samples from control in PCA plots (29). Similarly, such division was also seen between control groups (GI and CO) and PCB treatment groups (PCBs and PCBs+ACN) in the current study. PCB 126 has been shown to lower the microbial species richness and evenness in mice (14), in agreement with the findings regarding species richness (Chao 1 index) in the present work. The specific taxa changes after PCBs exposure varies in different mouse model studies. For example, PCB 126 exposure increased the relative abundance of *Helicobacter*, *Ruminiclostridium*, and *Rikenella* while lowering the relative abundance of *Allobaculum* in female mice (28). Rats exposed to PCB 126 decreased the bacterial counts of *Lactobacilli* and increased the counts of *Enterobacteriaceae* (67). Non-dioxin-like PCB 153 exposure depleted *Actinobacteria* and enriched *Bifidobacterium* and *Coriobacteriales* in female mice (29). In the present study, bacteria such as *Helicobacter*, *Allobaculum*, and *Lactobacilli* were not detected in the human fecal samples of this study. PCB exposure was associated with a decreased relative abundance of *Streptococcus, Veillonella*, *Holdemanella*, and *Dialister* and an increased relative abundance of *Akkermansia*, *Bifidobacterium*, and *Eggerthella*. Although decreased abundance of these taxa has not been described previously with PCB exposure, reduced relative abundance of *Veillonella* was noted in infants exposed to polybrominated diphenyl ethers from breast milk (68). In the current study, an increased abundance of *Akkermansia* and *Bifidobacterium* was noted in response to PCB exposure. A similar enrichment of *Akkermansia* and *Bifidobacterium* has also been observed in mice exposed to PCBs (14, 29). Despite their presumed health-promoting properties (69, 70), an increased abundance of these species may not always benefit the host. *Akkermansia* is a gram-negative, mucin-degrading bacteria (71). Mouse studies have shown that an increased abundance of *Akkermansia* is associated with a thinner mucin layer and a reduced number of goblet cells in the cecum, which can disrupt gut barrier integrity and so increase the risk of inflammation (71–73). An overgrowth of *Akkermansia* has also been observed in fecal samples from type 2 diabetes subjects (74) and patients receiving broad-spectrum antibiotic therapy (75). An increased abundance of *Bifidobacterium* has been reported in patients with irritable bowel disease (76). In the present study, PCB exposure was associated with an increased abundance of *Eggerthella* that has been related to an increased risk of colonic infection and colon cancer (77). Although *Proteobacteria* was the least dominant phyla for all treatments in the present study, the relative abundance of this phylum decreased in both PCBs and PCBs+ACN over the 12 h digestion period, which demonstrates that such acute exposure can lead to diminished and loss of growth of the *Proteobacteria* phylum. To date, the associations between PCB exposure and human gut microbiota has only been previously examined in two population studies. PCB exposure from breastmilk has been associated with reduced diversity of gut microbiota composition and decreased abundance of *Lactobacillus* spp. in 1 month-old infants (68). Prenatal PCB exposure has been associated with an increased relative abundance of *Propionibacteriales* and *Propionibacteriaceae* in mid-childhood (68). The above taxa were not predominant taxa in the fecal samples in the present work, which might be due to different gut microbiota profiles of infants and children versus the adult sample used in our study.

Anthocyanins did not counteract the PCB-mediated reduction in species richness and altered gut microbiota structure. The beta diversity data showed that the PCBs+ACN samples had a similar reduction in species richness and diversity as compared fecal samples exposed to PCBs alone. The ACN treatment, however, did mitigate against PCB perturbation of the abundance of specific taxa. ACNs suppressed PCB-induced overgrowth of *Akkermansia, Eggerthella*, and *Bifidobacterium.* Also, an increase in the relative abundance of *Faecalibacterium* was observed in PCB samples treated with ACNs, which is in agreement with observations of an increased relative abundance of *Faecalibacterium* in mice treated with ACN-rich black raspberry powder (78). *Faecalibacterium* are beneficial bacteria that have been implicated in promoting the gut environment by improving barrier function and controlling inflammation (79).

Polychlorinated biphenyls inhibited the production of total SCFAs, modulated mainly via a reduction in acetate content. Iszatt et al. (68) reported that the impact of PCB exposure via breastmilk on infant fecal SCFAs was congener specific. Non-dioxin like PCB 209 was associated with lower acetate content whereas dioxin-like PCB 167 was associated with increased acetate and propionate concentrations (31). On the other hand, *in vitro* fermentation studies involving mouse fecal matter showed a reduction in succinate production and enhanced propionate production with no change in acetate content after 48 h exposure to 2 μM of PCB 126 (80). The contrasting results to the present study could be due to species-related differences in microbial SCFA production. Notably, lactate (69%) is the most abundant SCFA in mice, followed by acetate (25%), propionate (3%) and butyrate (3%) (81) whereas acetate is the most abundant SCFA in human fecal matter, followed by propionate and butyrate (22). The acetate concentrations (42.3 ± 11 mM) in our control samples (collected from an Asian female) are in the range of previously reported human fecal acetate values (39.9–56.1 mM) from an Asian cohort (22). Likewise, the propionate (22.9 ± 6 mM) and butyrate (25 ± 11 mM) concentrations in the current study are also similar to propionate (12.8–23.6 mM) and butyrate (12.9–19 mM) concentrations in human fecal samples from an Asian cohort, respectively (22). The decreased level of propionate and butyrate in response to PCB exposure might be partly related to the reduced abundance of butyrate- and propionate-producing *Holdemanella and Dialister* (82), and propionate-producing *Veillonella* in the PCB-treated samples (22).

The ACN-rich treatment positively modulated the production of SCFAs, especially acetate, to mitigate against inhibition of SCFAs production by PCBs. In contrast to acetate, the butyrate content (7 mM) in the ACN alone treatment was reduced at 8 h digestion compared to GI and CO; however, the butyrate content (14 mM) at the longer digestion period of 12 h showed no significant difference as compared to the control GI group. Also, the lower propionate (15 mM) and butyrate (14 mM) concentrations of the ACN treatment at the 12 h digestion still fall within the physiological ranges in human fecal samples as previously described above (22). The batch culture fermentation system is a common model to study the metabolic profile of SCFAs produced from gut microbiota fermentation of prebiotics (42). The *in vitro* fermentation model using human fecal matter has shown that sweet potato ACNs increased total SCFA and acetate concentrations, which coincides with the present findings (40). Animal model studies have also shown a similar induction of fecal SCFA production following ACN treatment (38, 39). Mixtures of acetate, propionate and butyrate have been indicated to provide carbon sources to enhance microbial dechlorination of PCBs in carbon-limited sediment slurries, which could limit toxicity of PCBs in the environment (83). It is conceivable that SCFAs could play a role in gut microbial PCB dechlorination and detoxification, which requires further investigation.

The computer-controlled batch culture fermentation system was used in this study. This fermentation system is a valid and convenient model to assess the interactions between dietary components such as complex carbohydrates or resistant starch and gut microbiota composition (40, 42). One limitation is that there is no membrane or intestinal epithelium in the reactor of the batch culture fermentation model to enable absorption of digestible materials or microbial metabolites such as SCFAs during fermentation (84). During digestion, ACN content has been shown to be stable going from the oral to gastric phases, allowing the majority of ACNs to transit to the intestinal digestion and colonic fermentation phases (85). ACNs, however, have a low bioavailability as only 5–10% are absorbed in the small intestine (38). The majority of ACNs reach the colon intact to exert prebiotic effects on gut microbiota that can promote colonic health (38). The other limitation of this study is that the collected fecal sample used in the batch culture fermentation system came from a single donor. Both individual and pooled fecal samples have been indicated to have their own inherent limitations in batch culture fermentation studies (86, 87). The use of mixed fecal samples from different individuals could minimize the inter-individual diversity in the composition of human gut microbiota (86). On the other hand, Roberts et al. (87) suggested that the fermentation profiles of mixed fecal samples only reflect competitively dominant bacterial species from one or more donors (niche exclusion principle) rather than a physiological bacterial ecosystem. Van den Abbeele et al. (88) compared the impact of wheat-derived prebiotic fiber and inulin on microbial profiles and SCFA production using inoculation of pooled samples in the dynamic *in vitro* TNO colon model (TIM-2) versus inoculation of a fecal sample from a single donor in the Simulator of the Human Intestinal Microbial Ecosystem (SHIME). Their results demonstrated that the composition of the bacterial community profiles and SCFA production were similar between the two experimental settings (88). Thus, the functional differences between the use of single donor versus multiple donors for batch culture fermentation studies are unclear, which has been attributed to major functional overlaps of the microbiota among different individuals (86). Existing research evidence has suggested that inter-individual variability in host genotype and gut microbial metabolism of certain types of polyphenols could modulate their protective effects on chronic disorders such as neuroinflammation, cardiovascular disease and metabolic syndrome (89). To date, it is not known whether there exists inter-individual variability in the metabolism of the primary ACNs found in blue- or purple-fleshed potatoes, such as petunidin 3-p-coumaroylrutinoside-5-glucoside and malvidin 3-feruloylrutinoside-5-glucoside (37). Future studies could involve investigating whether inter-individual variability in gut microbiota composition can lead to variations in the health benefits of ACN-rich potato meals, including their protective effects against PCB-induced gut microbiota dysbiosis.

## 5. Conclusion

Short-term exposure to PCBs disrupted gut microbiota composition and diversity and inhibited SCFA production. ACN-rich potato treatment protected against PCB-induced diminishment of SCFAs and mitigated against changes in the relative abundances of specific taxa. To our knowledge, this is the first study investigating the impact of PCBs on human associated gut microbiota profile and production of SCFAs in a batch culture fermentation system. This is also the first study assessing the protective effects of ACN-rich potato meals on PCB-induced disruption of gut microbiota and the production of SCFAs.

## Data availability statement

The datasets presented in this study can be found in online repositories. The names of the repository/repositories and accession number(s) can be found below: https://www.ncbi.nlm.nih.gov/, PRJNA914676.

## Ethics statement

Ethical review and approval was not required for the study on human participants in accordance with the local legislation and institutional requirements. Written informed consent for participation was not required for this study in accordance with the national legislation and the institutional requirements.

## Author contributions

FL designed the study, carried out all the experiments, performed the statistical analysis, and wrote the first draft of the manuscript. CWM helped with fecal samples DNA extraction and statistical analysis and provided edits to the manuscript. JT performed the statistical analysis and edited the manuscript. SK initiated the original idea of the study, supervised all the aspects of the study, and helped review the manuscript. MMI was involved in the method development and review of the manuscript. All authors contributed to the article and approved the submitted version.

## References

[1] Agency for Toxic Substances and Disease Registry [ATSDR]. *Toxicological profile for Polychlorinated Biphenyls (PCBs).* Atlanta: U.S. Department of Health and Human Services (2000). p. 1–4. 36888731

[2] Quinete N Schettgen T Bertram J Kraus T. Occurrence and distribution of PCB metabolites in blood and their potential health effects in humans: a review. *Environ Sci Pollut Res.* (2014) 21:11951–72. 10.1007/s11356-014-3136-9 24943885

[3] van den Berg M Kypke K Kotz A Tritscher A Lee S Magulova K WHO/UNEP global surveys of PCDDs, PCDFs, PCBs and DDTs in human milk and benefit–risk evaluation of breastfeeding. *Arch Toxicol.* (2017) 91:83–96. 10.1007/s00204-016-1802-z 27438348 PMC5225187

[4] Singh K Karthikeyan S Vladisavljevic D St-Amand A Chan H. Factors associated with plasma concentrations of polychlorinated biphenyls (PCBs) and dichlorodiphenyldichloroethylene (p,p’-DDE) in the Canadian population. *Int J Environ Health Res.* (2019) 29:326–47. 30431336 10.1080/09603123.2018.1543799

[5] Morales-Suarez-Varela M Lopez Santana N Marti Requena P Beser Santos M Peraita-Costa I Llopis-Gonzalez A. Estimation of daily intake of polychlorinated biphenyls not similar to dioxins (NDL-PCB) from fish consumption in Spain in different population groups. *Public Health Nutr.* (2018) 21:2959–68. 30180916 10.1017/S1368980018002033 PMC10260906

[6] Habibullah-Al-Mamun M Ahmed M Islam M Hossain A Tokumura M Masunaga S. Polychlorinated biphenyls (PCBs) in commonly consumed seafood from the coastal area of Bangladesh: occurrence, distribution, and human health implications. *Environ Sci Pollut Res Int.* (2019) 26:1355–69. 10.1007/s11356-018-3671-x 30426367

[7] Newsome W Davies D Sun W. Residues of polychlorinated biphenyls (PCB) in fatty foods of the Canadian diet. *Food Addit Contam.* (1998) 15:19–29. 9534869 10.1080/02652039809374596

[8] Heindel J Blumberg B Cave M Machtinger R Mantovani A Mendez M Metabolism disrupting chemicals and metabolic disorders. *Reprod Toxicol.* (2017) 68:3–33. 27760374 10.1016/j.reprotox.2016.10.001 PMC5365353

[9] Shan Q Li H Chen N Qu F Guo J. Understanding the multiple effects of PCBs on lipid metabolism. *Diabetes Metab Syndr Obes.* (2020) 13:3691–702. 33116719 10.2147/DMSO.S264851 PMC7568599

[10] Wolf K Bongaerts B Schneider A Huth C Meisinger C Peters A Persistent organic pollutants and the incidence of type 2 diabetes in the CARLA and KORA cohort studies. *Environ Int.* (2019) 129:221–8. 10.1016/j.envint.2019.05.030 31132656

[11] Clair H Pinkston C Rai S Pavuk M Dutton N Brock G Liver disease in a residential cohort with elevated polychlorinated biphenyl exposures. *Toxicol Sci.* (2018) 164:39–49. 10.1093/toxsci/kfy076 29684222 PMC6016643

[12] Ghosh S Murinova L Trnovec T Loffredo C Washington K Mitra P Biomarkers linking PCB exposure and obesity. *Curr Pharm Biotechnol.* (2014) 15:1058–68. 25420728 10.2174/1389201015666141122203509 PMC4292903

[13] Rohm T Meier D Olefsky J Donath M. Inflammation in obesity, diabetes, and related disorders. *Immunity.* (2022) 55:31–55. 10.1016/j.immuni.2021.12.013 35021057 PMC8773457

[14] Petriello M Hoffman J Vsevolozhskaya O Morris A Hennig B. Dioxin-like PCB 126 increases intestinal inflammation and disrupts gut microbiota and metabolic homeostasis. *Environ Pollut.* (2018) 242:1022–32. 10.1016/j.envpol.2018.07.039 30373033 PMC6211811

[15] Phillips M Dheer R Santaolalla R Davies J Burgueno J Lang J Intestinal exposure to PCB 153 induces inflammation via the ATM/NEMO pathway. *Toxicol Appl Pharmacol.* (2018) 339:24–33. 10.1016/j.taap.2017.11.027 29197519 PMC6021014

[16] Qamar A Waheed J Ghulam Mohyuddin S Chen Z Kang D Li Z The status of polychlorinated biphenyls (PCBs) extract from zhanjiang mangrove sediments and the effects on tissue structure and inflammatory cytokines in zebrafish liver. *Bull Environ Contam Toxicol.* (2022) 108:890–900. 10.1007/s00128-021-03439-6 35133448

[17] Kho Z Lal S. The human gut microbiome - a potential controller of wellness and disease. *Front Microbiol.* (2018) 9:1835. 10.3389/fmicb.2018.01835 30154767 PMC6102370

[18] Holmes E Li J Athanasiou T Ashrafian H Nicholson J. Understanding the role of gut microbiome-host metabolic signal disruption in health and disease. *Trends Microbiol.* (2011) 19:349–59. 10.1016/j.tim.2011.05.006 21684749

[19] Singh R Chang H Yan D Lee K Ucmak D Wong K Influence of diet on the gut microbiome and implications for human health. *J Transl Med.* (2017) 15:73. 10.1186/s12967-017-1175-y PMC5385025 28388917

[20] Claus S Guillou H Ellero-Simatos S. The gut microbiota: a major player in the toxicity of environmental pollutants? *NPJ Biofilms Microbiomes.* (2016) 2:16003. 10.1038/npjbiofilms.2016.3 PMC5515271 28721242

[21] Riccio P Rossano R. The human gut microbiota is neither an organ nor a commensal. *FEBS Lett.* (2020) 594:3262–71. 33011965 10.1002/1873-3468.13946

[22] Parada Venegas D De la Fuente M Landskron G Gonzalez M Quera R Dijkstra G Short chain fatty acids (SCFAs)-mediated gut epithelial and immune regulation and its relevance for inflammatory bowel diseases. *Front Immunol.* (2019) 10:277. 10.3389/fimmu.2019.00277 30915065 PMC6421268

[23] Akhtar M Chen Y Ma Z Zhang X Shi D Khan J Gut microbiota-derived short chain fatty acids are potential mediators in gut inflammation. *Anim Nutr.* (2022) 8:350–60. 35510031 10.1016/j.aninu.2021.11.005 PMC9040132

[24] Gasaly N Hermoso M Gotteland M. Butyrate and the fine-tuning of colonic homeostasis: implication for inflammatory bowel diseases. *Int J Mol Sci.* (2021) 22:3061. 10.3390/ijms22063061 33802759 PMC8002420

[25] Chen G Ran X Li B Li Y He D Huang B Sodium butyrate inhibits inflammation and maintains epithelium barrier integrity in a TNBS-induced inflammatory bowel disease mice model. *EBioMedicine.* (2018) 30:317–25. 10.1016/j.ebiom.2018.03.030 29627390 PMC5952406

[26] Deleu S Machiels K Raes J Verbeke K Vermeire S. Short chain fatty acids and its producing organisms: an overlooked therapy for IBD? *EBioMedicine.* (2021) 66:103293. 10.1016/j.ebiom.2021.103293 33813134 PMC8047503

[27] Choi J Eum S Rampersaud E Daunert S Abreu M Toborek M. Exercise attenuates PCB-induced changes in the mouse gut microbiome. *Environ Health Persp.* (2013) 121:725–30. 10.1289/ehp.1306534 23632211 PMC3672930

[28] Chi Y Lin Y Lu Y Huang Q Ye G Dong S. Gut microbiota dysbiosis correlates with a low-dose PCB126-induced dyslipidemia and non-alcoholic fatty liver disease. *Sci Total Environ.* (2019) 653:274–82. 10.1016/j.scitotenv.2018.10.387 30412872

[29] Min L Chi Y Dong S. Gut microbiota health closely associates with PCB153-derived risk of host diseases. *Ecotoxicol Environ Saf.* (2020) 203:111041. 10.1016/j.ecoenv.2020.111041 32888612

[30] Li T Tian D Lu M Wang B Li J Xu B Gut microbiota dysbiosis induced by polychlorinated biphenyl 126 contributes to increased brain proinflammatory cytokines: landscapes from the gut-brain axis and fecal microbiota transplantation. *Ecotoxicol Environ Saf.* (2022) 241:113726. 10.1016/j.ecoenv.2022.113726 35691195

[31] Tian Y Rimal B Gui W Koo I Smith P Yokoyama S Early life polychlorinated biphenyl 126 exposure disrupts gut microbiota and metabolic homeostasis in mice fed with high-fat diet in adulthood. *Metabolites.* (2022) 12:894. 10.3390/metabo12100894 36295797 PMC9609008

[32] Lim J Li X Lehmler H Wang D Gu H Cui J. Gut microbiome critically impacts PCB-induced changes in metabolic fingerprints and the hepatic transcriptome in mice. *Toxicol Sci.* (2020) 177:168–87. 10.1093/toxsci/kfaa090 32544245 PMC7553702

[33] Ashaolu T Ashaolu J Adeyeye S. Fermentation of prebiotics by human colonic microbiota in vitro and short-chain fatty acids production: a critical review. *J Appl Microbiol.* (2021) 130:677–87. 10.1111/jam.14843 32892434

[34] Igwe E Charlton K Probst Y Kent K Netzel ME. A systematic literature review of the effect of anthocyanins on gut microbiota populations. *J Hum Nutr Diet.* (2019) 32:53–62. 10.1111/jhn.12582 29984532

[35] Blesso C. Dietary anthocyanins and human health. *Nutrients.* (2019) 11:2107. 10.3390/nu11092107 PMC6770874 31491856

[36] Burgos G Zum Felde T Andre C Kubow S. The potato and its contribution to the human diet and health. In: Campos H Ortiz O editors. *The potato crop: its agricultural, nutritional and social contribution to humankind.* Cham: Springer International Publishing (2020). p. 37–74.

[37] Oertel A Matros A Hartmann A Arapitsas P Dehmer K Martens S Metabolite profiling of red and blue potatoes revealed cultivar and tissue specific patterns for anthocyanins and other polyphenols. *Planta.* (2017) 246:281–97. 10.1007/s00425-017-2718-4 28664422

[38] Jamar G Estadella D Pisani L. Contribution of anthocyanin-rich foods in obesity control through gut microbiota interactions. *Biofactors.* (2017) 43:507–16. 10.1002/biof.1365 28504479

[39] Faria A Fernandes I Norberto S Mateus N Calhau C. Interplay between anthocyanins and gut microbiota. *J Agric Food Chem.* (2014) 62: 6898–902. 24915058 10.1021/jf501808a

[40] Zhang X Yang Y Wu Z Weng P. The modulatory effect of anthocyanins from purple sweet potato on human intestinal microbiota in vitro. *J Agric Food Chem.* (2016) 64:2582–90. 10.1021/acs.jafc.6b00586 26975278

[41] Jennings A Koch M Jensen M Bang C Kassubek J Muller H The role of the gut microbiome in the association between habitual anthocyanin intake and visceral abdominal fat in population-level analysis. *Am J Clin Nutr.* (2020) 111:340–50. 10.1093/ajcn/nqz299 31826255 PMC6997102

[42] Payne A Zihler A Chassard C Lacroix C. Advances and perspectives in in vitro human gut fermentation modeling. *Trends Biotechnol.* (2012) 30:17–25. 21764163 10.1016/j.tibtech.2011.06.011

[43] Burgos G Amoros W Muñoa L Sosa P Cayhualla E Sanchez C Total phenolic, total anthocyanin and phenolic acid concentrations and antioxidant activity of purple-fleshed potatoes as affected by boiling. *J Food Composit Anal.* (2013) 30:6–12.

[44] Lee S Oh S Hwang I Kim H Woo K Woo S Antioxidant contents and antioxidant activities of white and colored potatoes (*Solanum tuberosum* L.). *Prev Nutr Food Sci.* (2016) 21:110–6. 10.3746/pnf.2016.21.2.110 27390727 PMC4935237

[45] Larder C Abergel M Kubow S Donnelly D. Freeze-drying affects the starch digestibility of cooked potato tubers. *Food Res Int.* (2018) 103:208–14. 10.1016/j.foodres.2017.10.034 29389607

[46] Gaisawat M Iskandar M MacPherson C Tompkins T Kubow S. Probiotic supplementation is associated with increased antioxidant capacity and copper chelation in C. difficile-infected fecal water. *Nutrients.* (2019) 11:2007. 10.3390/nu11092007 31454897 PMC6769851

[47] Tzounis X Vulevic J Kuhnle G George T Leonczak J Gibson G Flavanol monomer-induced changes to the human faecal microflora. *Br J Nutr.* (2008) 99:782–92. 10.1017/S0007114507853384 17977475

[48] Molly K Woestyne M Smet I Verstraete W. Validation of the simulator of the human intestinal microbial ecosystem (SHIME) reactor using microorganism-associated activities. *Microb Ecol Health Dis.* (1994) 7:191–200.

[49] Wassermann M Wassermann D Cucos S Miller H. World PCBs map: storage and effects in man and his biologic environment in the 1970s. *Ann NY Acad Sci.* (1979) 320:69–124. 10.1111/j.1749-6632.1979.tb13137.x 110205

[50] Kirkwood Z Millar B Downey D Moore J. Antimicrobial effect of dimethyl sulfoxide and N, N-Dimethylformamide on Mycobacterium abscessus: implications for antimicrobial susceptibility testing. *Int J Mycobacteriol.* (2018) 7:134–6. 10.4103/ijmy.ijmy_35_18 29900888

[51] Reyes L Miller J Cisneros-Zevallos L. Antioxidant capacity, anthocyanins and total phenolics in purple-and red-fleshed potato (*Solanum tuberosum* L.) genotypes. *Am J Potato Res.* (2005) 82:271–7.

[52] Decker E Ferruzzi M. Innovations in food chemistry and processing to enhance the nutrient profile of the white potato in all forms. *Adv Nutr.* (2013) 4:345S–50S. 10.3945/an.112.003574 23674803 PMC3650506

[53] Gaisawat M MacPherson C Tremblay J Piano A Iskandar M Tompkins T Probiotic supplementation in a clostridium difficile-infected gastrointestinal model is associated with restoring metabolic function of microbiota. *Microorganisms.* (2019) 8:60. 10.3390/microorganisms8010060 31905795 PMC7023328

[54] Sadeghi Ekbatan S Sleno L Sabally K Khairallah J Azadi B Rodes L Biotransformation of polyphenols in a dynamic multistage gastrointestinal model. *Food Chem.* (2016) 204:453–62. 10.1016/j.foodchem.2016.02.140 26988524

[55] MacPherson C Mathieu O Tremblay J Champagne J Nantel A Girard S Gut bacterial microbiota and its resistome rapidly recover to basal state levels after short-term amoxicillin-clavulanic acid treatment in healthy adults. *Sci Rep.* (2018) 8:11192. 10.1038/s41598-018-29229-5 30046129 PMC6060159

[56] Tremblay J Yergeau E. Systematic processing of ribosomal RNA gene amplicon sequencing data. *Gigascience.* (2019) 8:giz146. 10.1093/gigascience/giz146 PMC6901069 31816087

[57] Callahan B McMurdie P Rosen M Han A Johnson A Holmes S. DADA2: high-resolution sample inference from Illumina amplicon data. *Nat Methods.* (2016) 13:581–3. 10.1038/nmeth.3869 27214047 PMC4927377

[58] Edgar R Haas B Clemente J Quince C Knight R. UCHIME improves sensitivity and speed of chimera detection. *Bioinformatics.* (2011) 27:2194–200. 10.1093/bioinformatics/btr381 21700674 PMC3150044

[59] Wang Q Garrity G Tiedje J Cole J. Naïve bayesian classifier for rapid assignment of rRNA sequences into the new bacterial taxonomy. *Appl Environ Microbiol.* (2007) 73:5261–7. 17586664 10.1128/AEM.00062-07 PMC1950982

[60] Quast C Pruesse E Yilmaz P Gerken J Schweer T Yarza P The SILVA ribosomal RNA gene database project: improved data processing and web-based tools. *Nucleic Acids Res.* (2013) 41:D590–6. 10.1093/nar/gks1219 23193283 PMC3531112

[61] DeSantis T Hugenholtz P Larsen N Rojas M Brodie E Keller K Greengenes, a chimera-checked 16S rRNA gene database and workbench compatible with ARB. *Appl Environ Microbiol.* (2006) 72:5069–72. 10.1128/AEM.03006-05 16820507 PMC1489311

[62] Caporaso J Kuczynski J Stombaugh J Bittinger K Bushman F Costello E QIIME allows analysis of high-throughput community sequencing data. *Nat Methods.* (2010) 7:335–6. 20383131 10.1038/nmeth.f.303 PMC3156573

[63] Price M Dehal P Arkin A. FastTree 2–approximately maximum-likelihood trees for large alignments. *PLoS One.* (2010) 5:e9490. 10.1371/journal.pone.0009490 20224823 PMC2835736

[64] Kuczynski J Stombaugh J Walters WA Gonzalez A Caporaso JG Knight R. Using QIIME to analyze 16S rRNA gene sequences from microbial communities. *Curr Protoc Bioinformatics*. (2011) 10:10.7.1–7.20. 10.1002/0471250953.bi1007s36 22161565 PMC3249058

[65] Durden C Dong Q. RICHEST–a web server for richness estimation in biological data. *Bioinformation.* (2009) 3:296–8. 10.6026/97320630003296 19293995 PMC2655047

[66] Reese A Dunn R. Drivers of microbiome biodiversity: a review of general rules, feces, and ignorance. *mBio.* (2018) 9:e01294–18. 10.1128/mBio.01294-18 30065092 PMC6069118

[67] Kaoru Y. T, 2 Mitsuyuki SHIRAI,2 Tatsuya TAKIZAWA,2 Tadashi SHINODA,3 Toshio MASAOKA,2 Fumiaki AKAHORI2 and Hidetoshi MORITA. Effect of oral administration of 3,3′,4,4′,5-pentachlorobiphyenl on the intestinal microbiota of Sprague–Dawley rats. *Anim Sci J.* (2008) 79:391–400.

[68] Iszatt N Janssen S Lenters V Dahl C Stigum H Knight R Environmental toxicants in breast milk of Norwegian mothers and gut bacteria composition and metabolites in their infants at 1 month. *Microbiome.* (2019) 7:34. 10.1186/s40168-019-0645-2 30813950 PMC6393990

[69] O’Callaghan A van Sinderen D. Bifidobacteria and their role as members of the human gut microbiota. *Front Microbiol.* (2016) 7:925. 10.3389/fmicb.2016.00925 27379055 PMC4908950

[70] Naito Y Uchiyama K Takagi T. A next-generation beneficial microbe: akkermansia muciniphila. *J Clin Biochem Nutr.* (2018) 63:33–5. 30087541 10.3164/jcbn.18-57 PMC6064808

[71] Si J Kang H You H Ko G. Revisiting the role of Akkermansia muciniphila as a therapeutic bacterium. *Gut Microbes.* (2022) 14:2078619. 10.1080/19490976.2022.2078619 35613313 PMC9135416

[72] Ganesh B Klopfleisch R Loh G Blaut M. Commensal Akkermansia muciniphila exacerbates gut inflammation in *Salmonella* Typhimurium-infected gnotobiotic mice. *PLoS One.* (2013) 8:e74963. 10.1371/journal.pone.0074963 24040367 PMC3769299

[73] Hamilton M Boudry G Lemay D Raybould H. Changes in intestinal barrier function and gut microbiota in high-fat diet-fed rats are dynamic and region dependent. *Am J Physiol Gastrointest Liver Physiol.* (2015) 308:G840–51. 10.1152/ajpgi.00029.2015 25747351 PMC4437018

[74] Qin J Li Y Cai Z Li S Zhu J Zhang F A metagenome-wide association study of gut microbiota in type 2 diabetes. *Nature.* (2012) 490: 55–60. 23023125 10.1038/nature11450

[75] Dubourg G Lagier J Armougom F Robert C Audoly G Papazian L High-level colonisation of the human gut by Verrucomicrobia following broad-spectrum antibiotic treatment. *Int J Antimicrob Agents.* (2013) 41:149–55. 10.1016/j.ijantimicag.2012.10.012 23294932

[76] Wang W Chen L Zhou R Wang X Song L Huang S Increased proportions of Bifidobacterium and the Lactobacillus group and loss of butyrate-producing bacteria in inflammatory bowel disease. *J Clin Microbiol.* (2014) 52:398–406. 10.1128/JCM.01500-13 24478468 PMC3911339

[77] Gardiner B Tai A Kotsanas D Francis M Roberts S Ballard S Clinical and microbiological characteristics of Eggerthella lenta bacteremia. *J Clin Microbiol.* (2015) 53:626–35. 25520446 10.1128/JCM.02926-14 PMC4298500

[78] Chen L Jiang B Zhong C Guo J Zhang L Mu T Chemoprevention of colorectal cancer by black raspberry anthocyanins involved the modulation of gut microbiota and SFRP2 demethylation. *Carcinogenesis.* (2018) 39:471–81. 10.1093/carcin/bgy009 29361151

[79] Xu J Liang R Zhang W Tian K Li J Chen X Faecalibacterium prausnitzii-derived microbial anti-inflammatory molecule regulates intestinal integrity in diabetes mellitus mice via modulating tight junction protein expression. *J Diabetes.* (2020) 12:224–36. 10.1111/1753-0407.12986 31503404 PMC7064962

[80] Hoffman J Flythe M Hennig B. Environmental pollutant-mediated disruption of gut microbial metabolism of the prebiotic inulin. *Anaerobe.* (2019) 55:96–102. 10.1016/j.anaerobe.2018.11.008 30447394 PMC6481639

[81] Nagpal R Wang S Solberg Woods L Seshie O Chung S Shively C Comparative microbiome signatures and short-chain fatty acids in mouse, rat, non-human primate, and human feces. *Front Microbiol.* (2018) 9:2897. 10.3389/fmicb.2018.02897 30555441 PMC6283898

[82] Louis P Flint H. Formation of propionate and butyrate by the human colonic microbiota. *Environ Microbiol.* (2017) 19:29–41. 27928878 10.1111/1462-2920.13589

[83] Wiegel J Wu Q. Microbial reductive dehalogenation of polychlorinated biphenyls. *FEMS Microbiol Ecol.* (2000) 32:1–15. 10779614 10.1111/j.1574-6941.2000.tb00693.x

[84] Aura A Maukonen J. One compartment fermentation model. In: Verhoeckx K Cotter P López-Expósito I Kleiveland C Lea T Mackie A editors. *The impact of food bioactives on health: in vitro and ex vivo models.* Cham: Springer International Publishing (2015). p. 281–92. 29787039

[85] Eker M Aaby K Budic-Leto I Brncic S El S Karakaya S A Review of factors affecting anthocyanin bioavailability: possible implications for the inter-individual variability. *Foods.* (2019) 9:2. 10.3390/foods9010002 31861362 PMC7023094

[86] Aguirre M Venema K. Challenges in simulating the human gut for understanding the role of the microbiota in obesity. *Benef Microbes.* (2017) 8:31–53. 10.3920/BM2016.0113 27903093

[87] Roberts K Allen-Vercoe E Williams S Graham T Cui S. Comparative study of the in vitro fermentative characteristics of fenugreek gum, white bread and bread with fenugreek gum using human faecal microbes. *Bioact Carbohydr Diet Fibre.* (2015) 5:116–24.

[88] Van den Abbeele P Venema K Van de Wiele T Verstraete W Possemiers S. Different human gut models reveal the distinct fermentation patterns of Arabinoxylan versus inulin. *J Agric Food Chem.* (2013) 61:9819–27. 10.1021/jf4021784 24028202

[89] Zhang B Zhang Y Xing X Wang S. Health benefits of dietary polyphenols: insight into interindividual variability in absorption and metabolism. *Curr Opin Food Sci.* (2022) 48:100941.

