# Supplementary material for: Anthocyanin-rich blue potato meals protect against polychlorinated biphenyl-mediated disruption of short-chain fatty acid production and gut microbiota profiles in a simulated human digestion model
Source: Front Nutr. 2023 May 31;10:1130841. doi: 10.3389/fnut.2023.1130841 (PMC10266533; doi:10.3389/fnut.2023.1130841)
Supplement: Supplementary file 1 [file Data_Sheet_1.docx]

Supplementary Material

**Anthocyanin protection against polychlorinated biphenyls (PCBs)-mediated disruption in short-chain fatty acid (SCFA) production in a simulated human digestion model**

**Fang Lu^1^, Chad W. MacPherson^2^, Julien Tremblay^3^, Michèle M. Iskandar^1^, and Stan Kubow^1*^**

^1^ School of Human Nutrition, McGill University, Ste-Anne-de-Bellevue, QC, Canada

^2^ NutraPharma Consulting Services, Inc., Montréal, QC, Canada

^3^ Energy, Mining and Environment, National Research Council Canada, Montréal, QC, Canada

*** Correspondence:**Dr. Stan Kubow
stankubow@mcgill.ca

Keywords: polychlorinated biphenyl 153, polychlorinated biphenyl 126, gut microbiota, anthocyanins, short-chain fatty acids, simulated gut model, 16S rRNA gene amplicon sequencing, V3-V4 hypervariable regions

## 1. Supplementary Figures

**Supplementary Figure 1.** The concentrations of the six individual branched chain fatty acids (isobutyrate, isovaleric acid, valeric acid) and medium chain fatty acids (isocaproic acid, caproic acid and heptanoic acid) in fecal water under different treatments. Data is represented by means ± SEM. Two-way ANOVA followed by Tukey HSD test was used to assess for significant differences. Treatments not sharing common letters are significantly different (*p*<0.05) at the same digestion timepoints. The symbols # , $ represent significant (*p*<0.05) differences over the 12 h digestion time within the same treatment. Bars not sharing same letters (a, b, c) are statistically different between treatments (*p*<0.05).

**Supplementary Figure 2.** The relative abundance of major individual bacteria at genus level in fecal samples under different treatments. Data is represented by means ± SEM. Two-way ANOVA followed by Tukey HSD test was used to assess for significant differences. Treatments not sharing common letters (a, b, c) have significantly different (*p*<0.05) relative abundance with all timepoints jointly combined.
